# Red Cell Storage Duration Does Not Affect Outcome after Massive Blood Transfusion in Trauma and Nontrauma Patients: A Retrospective Analysis of 305 Patients

**DOI:** 10.1155/2017/3718615

**Published:** 2017-05-14

**Authors:** Alexander Bautista, Theodore B. Wright, Janice Meany, Sunitha K. Kandadai, Benjamin Brown, Kareim Khalafalla, Saeed Hashem, Jason W. Smith, Tayyeb M. Ayyoubi, Jarrod E. Dalton, Anupama Wadhwa, Daniel I. Sessler, Detlef Obal

**Affiliations:** ^1^Department of Anaesthesiology and Perioperative Medicine, University of Louisville, Louisville, KY, USA; ^2^Blood Bank University Hospital, Institute of Pathology, University of Louisville, Louisville, KY, USA; ^3^Trauma Institute, Department of Surgery, University of Louisville, Louisville, KY, USA; ^4^Department of Quantitative Health Sciences, Cleveland Clinic, Cleveland, OH, USA; ^5^Department of Outcomes Research, Anaesthesiology Institute, Cleveland Clinic, Cleveland, OH, USA

## Abstract

**Background:**

Prolonged storage of packed red blood cells (PRBCs) may increase morbidity and mortality, and patients having massive transfusion might be especially susceptible. We therefore tested the hypothesis that prolonged storage increases mortality in patients receiving massive transfusion after trauma or nontrauma surgery. Secondarily, we considered the extent to which storage effects differ for trauma and nontrauma surgery.

**Methods:**

We considered surgical patients given more than 10 units of PRBC within 24 hours and evaluated the relationship between mean PRBC storage duration and in-hospital mortality using multivariable logistic regression. Potential nonlinearities in the relationship were assessed via restricted cubic splines. The secondary hypothesis was evaluated by considering whether there was an interaction between the type of surgery (trauma versus nontrauma) and the effect of storage duration on outcomes.

**Results:**

305 patients were given a total of 8,046 units of PRBCs, with duration ranging from 8 to 36 days (mean ± SD: 22 ± 6 days). The odds ratio [95% confidence interval (CI)] for in-hospital mortality corresponding to a one-day in mean PRBC storage duration was 0.99 (0.95, 1.03, *P* = 0.77). The relationship did not differ for trauma and nontrauma patients (*P* = 0.75). Results were similar after adjusting for multiple potential confounders.

**Conclusions:**

Mortality after massive blood transfusion was no worse in patients transfused with PRBC stored for long periods. Trauma and nontrauma patients did not differ in their susceptibility to prolonged PRBC storage.

## 1. Background

Over 14 million units of blood products are transfused in the United States (U.S.) annually [1]. Massive blood transfusions are given to 3–5% [2] of the civilian and 8–10% [3] of the military trauma patient population. Patients requiring massive blood transfusion are at high risk for adverse clinical outcomes largely because of their serious trauma, but also as a direct consequence of receiving numerous blood products. Transfusions of blood products are associated with several complications and current evidence suggests that transfusions independently increase the risk of morbidity and death in critically ill patients [4–8], with mortality increasing linearly as a function of the amount of blood transfused. For example, a recent trauma registry analysis found that major blood loss constitutes an important prognostic factor for the survival [9] and the authors of the PROMMTT trial [10] demonstrated an adjusted odd ratio of 6-hour mortality for patients receiving ≥4 units within 30 minutes of 2.1 (95% confidence interval: 1.2–3.5). Overall, adverse consequences of transfusions add about $17 billion to United States healthcare costs which exceeds the costs of blood acquisition and transfusion combined [11].

Koch and colleagues identified a strong association between storage of red cells more than 14 days and major complications and mortality after coronary artery bypass graft (CABG) surgery [1]. The United States Food and Drug Administration allows storage of PRBCs for up to 42 days; however, it is well established that stored red cells undergo substantial biochemical and morphological changes during this period. Important dysfunction includes reduced oxygen delivery resulting from progressive decrease in 2,3-diphosphoglycerate (2,3-DPG) concentrations [12], reduced PRBC-dependent vasodilation [13] (for review see [14, 15]), decreased cell membrane deformability [16, 17] which potentially obstructs capillary flow, consumption of clotting factors, and activation of intravascular coagulation through PRBC derived microvesicles [18]. Prolonged PRBC storage is also thought to increase the risk of severe infection after CABG surgery [19].

Other studies, though, do not identify harm from transfusion of older blood. For example, Edgren and colleagues [20] analysed the Scandinavian Donations and Transfusions (SCANDAT) database and found that the initial trend towards a seven-day risk of death reduction diminishes within the 2-year follow-up and that transfusion of older blood is likely to contribute to less than 5% to excess mortality. Saager and colleagues similarly reported no relationship between prolonged median storage duration and mortality in a retrospective analysis of data from noncardiac surgery patients [21]. More importantly, two large randomized trials, RECESS and ABLE, concluded that PRBC storage duration did not affect the outcome after CABG surgery [22] or in critically ill patients [23].

Routine surgical patients, if given blood, usually receive just one or two units. Trauma patients differ in often experiencing major blood loss and consequently requiring large amounts of blood products. Trauma patients, along with nontrauma patients, who are given large amounts of blood (i.e., ≥10 units) may thus be especially susceptible to injury from blood that has been long stored.

Consistent with this theory, Zallen and colleagues performed a small prospective database analysis in trauma patients and concluded that multiorgan failure was more likely when patients were transfused with blood stored for 30 days than when stored for 24 days [24]. Two similar small studies suggest that prolonged blood storage duration is associated with an increased need [25] and increased duration of critical care [26]. Furthermore Weinberg and colleagues [27] included 176 trauma patients who were given at least one unit of blood within 24 hours after hospital arrival. Those given six units or more of PRBC, with at least three units stored for more than 14 days, had an odds ratio of dying of 7.8 (95% CI 2.3–26.3), twice the odds ratio of patients who received blood stored for less than 14 days. Previous published reports focused mainly on the impact of different storage durations in relative small numbers of transfused PRBCs (i.e., 5–10 units) on clinical outcome [24, 26, 28, 29].

We therefore tested the hypothesis that mean storage duration of transfused PRBC is associated with in-hospital mortality in patients given more than 10 units of PRBC within 24 hours. We also compared the relationship between storage duration and mortality in trauma and nontrauma patients who required massive blood transfusion for elective surgery. Specifically, we tested the secondary hypothesis that the relationship between blood storage duration and mortality is similar in trauma and nontrauma patients given massive amounts of blood.

## 2. Patients and Methods

The University of Louisville Hospital is a level-one trauma centre with about 3,000 trauma admissions/year. After the approval by the institutional review board at the University of Louisville, we performed this retrospective observational cohort study. Our analysis was restricted to patients who were given more than 10 units of PRBCs within a 24-hour surgical intervention according to the Hospital Blood Bank database. We include neither patients who died within six hours after starting massive blood transfusion and suffered from irreversible lethal injuries (i.e., gunshot to the head, open brain injuries) nor patients with more than 20% incomplete data or inadequate documentation within the medical records into our analysis. In addition to eligibility criteria for the study, we excluded patients for whom storage duration information was unavailable for >10% of their transfused PRBC units.

We accessed the University Hospital Information Management system to retrieve baseline demographic, perioperative, and outcome variables as listed in Table 1. Trauma scores and injury schemes were retrieved from the hospital's trauma database and reviewed by an attending trauma surgeon (J.S.).

Patients' medical records were reviewed from the time they arrived in the Emergency Department (ED, trauma patients) or the start of massive blood transfusion (nontrauma patients) until the time of discharge. We considered Emergency Department, Surgical and Anaesthesia, Laboratory, and Blood Bank records. Only initial surgical procedures were considered.

Information regarding storage duration of blood components, ABO blood type, and the time at which each product was released by the University of Louisville Hospital Blood Bank was collected from the same database. All PRBCs were leukoreduced by the blood supplier (usually the American Red Cross) and stored in AS-1 solution.

Preprocedure biochemistry and blood gas data were retrieved from either ED records or, if not available, from the initial intraoperative blood collection. Postprocedure biochemistry and haematological values were obtained from the final intraoperative or initial postanesthesia care unit blood samples.

### 2.1. Statistical Methods

Data analysis followed a stepwise approach: first, baseline potential confounding variables were assessed for balance using standard univariable numerical summaries across quartiles of observed mean PRBC storage duration.

To evaluate the principal hypothesis of the study, namely, that mean PRBC storage duration is associated with in-hospital mortality, we conducted both a univariable analysis and a (primary) multivariable analysis. Within our logistic regression models, patient mean PRBC storage duration was analysed as a continuous variable. Potential nonlinearities in the relationship between patient mean PRBC storage duration and mortality were assessed using a Chi-squared goodness of fit test, which compared a model that incorporated restricted cubic splines to a model that assumed a linear effect. The secondary hypotheses regarding the existence of differential relationships for trauma and nontrauma patients were evaluated by adding an interaction term between patient mean PRBC storage duration and an indicator for trauma to these models.

To construct the multivariable model, we used backward stepwise variable selection, starting with a “full” model that included the variables listed in Table 1 (excluding the indicator variables describing the type of trauma that occurred in lieu of the fact that we analysed trauma as a binary variable that was linearly dependent on these indicator variables) provided the variables were not >10% missing. Variable selection was implemented in a penalized fashion, based on Akaike's Information Criterion (AIC) [30]. By introducing a penalty proportional to each additional parameter in the model, the AIC encourages parsimonious models.

The potential confounding variables we considered were year of admission, age, female sex, patient blood type, along with a history of heart disease, pulmonary disease, renal disease, hypertension, diabetes mellitus, carcinoma, liver disease, tobacco use, alcohol use, or illicit drug use. We also considered preprocedural hemoglobins, heart rate, systolic blood pressure, blood pH, platelet count, international normalized ratio, prothrombin time, activated partial thromboplastin time, and number of transfused PRBC units. And finally, we also included duration of surgery. The multivariable models included only patients with complete information on these covariates.

R statistical software version 3.2.0 (The R Foundation for Statistical Computing, Vienna, Austria) for 64-bit Microsoft Windows operating system was used to perform all analyses. We used a Type I error rate of 5% for the evaluation of all hypotheses.

## 3. Results

**Figure 1:**
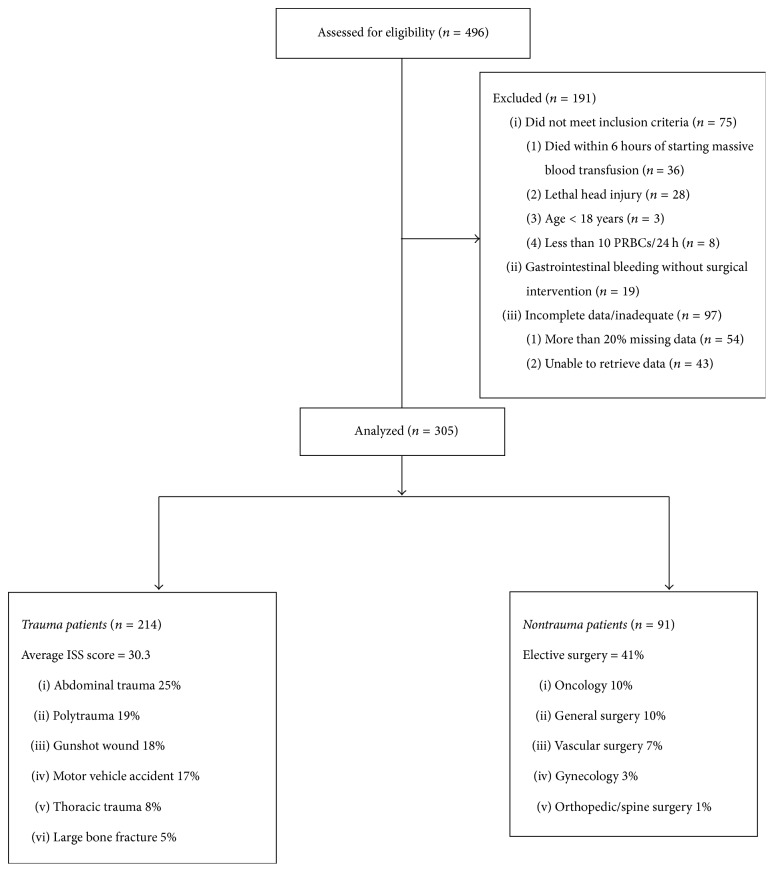
Enrolment scheme.

We identified 496 consecutive trauma and nontrauma patients who were given more than 10 units of PRBCs within 24 hours of surgery over a six-year period. 191 were excluded, mostly because patients died within six hours or because of missing data or failure to meet inclusion criteria. A detailed enrolment scheme is attached as Figure 1 and a summary of baseline characteristics for the four quartile groups of patient mean PRBC storage duration defined among the remaining 305 patients is given in Table 1. Baseline characteristics by type of surgery (trauma versus nontrauma) are presented in Table 2. With patients staying an average of 21 days (minimum: 1 day, maximum: 138 days) in the hospital, the overall in-hospital mortality was 101/305 (33.1%), including 23/90 (25.6%) of nontrauma patients and 76/213 (35.7%) of trauma patients.

**Figure 2:**
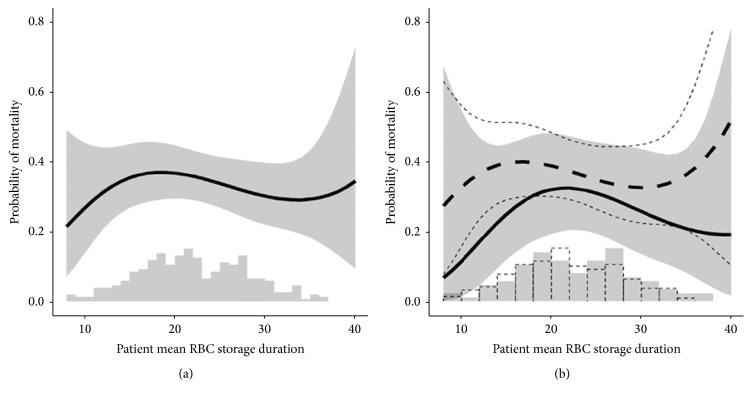
(a) Univariable relationship between patient mean red blood cell (RBC) storage duration and in-hospital mortality. Results presented as estimate and pointwise 95% confidence interval. A histogram of the observed values of patient mean RBC storage duration underlies the plot. The *P* value represents a Chi-squared test of association for a univariable logistic regression model which incorporated restricted cubic splines to allow for nonlinearities. (b) Univariable relationship between patient mean red blood cell (RBC) storage duration and in-hospital mortality, separately for trauma (dashed lines) and nontrauma (continuous line/shaded region) patients. Results presented as group-specific estimates and pointwise 95% confidence intervals. Histograms of the observed values of patient mean RBC storage duration underlie the plot. The lack of significance indicated by the *P* value in the figure indicates that our data did not support the hypothesis that the nature of the relationship differed among the groups (i.e., test for the interaction between group and patient mean RBC storage duration).

Overall, patient mean PRBC storage duration ranged from 8 to 37 days, with a mean ± standard deviation storage duration of 22 ± 6 days.  Figure 2(a) displays a histogram, as well as the estimated (nonlinear) relationship with in-hospital mortality, based on the univariable logistic regression model. The restricted cubic spline term which characterized the nonlinear relationship did not significantly improve the model fit (*P* = 0.78, Chi-squared goodness of fit test), suggesting that a linear approximation sufficed. Based on this simpler model, the odds ratio (95% confidence interval) for in-hospital mortality corresponding to a one-day increase in patient mean PRBC storage duration was estimated at 0.99 (0.95, 1.03, *P* = 0.77, Chi-squared test for model coefficients). No significant evidence of differential relationships between trauma and nontrauma patients was found (*P* = 0.87 for the interaction between trauma and patient mean PRBC storage duration; see Figure 2(b)).

Data from 259 patients were included in our multivariable modelling. The stepwise variable selection procedure identified age, renal disease, alcohol use, preprocedure blood pH, activated partial thromboplastin time, and number of units transfused as covariates for the final multivariable model. However, results were similar to those obtained from the univariable modelling. The adjusted odds ratio (95% CI) for patient mean PRBC storage duration was 0.98 (0.93, 1.04).

Secondary outcomes are summarized in Tables 3 and 4. There was no significant relationship between mean storage duration and in-hospital mortality. Patients who were given blood of the first and third quartile had a slightly (nonsignificantly) longer hospitalization, ICU stay, and mechanical ventilation than patients given blood from the second or fourth storage duration quartile.

About 22% of all patients suffered from disseminated intravascular coagulation, independent of blood storage duration, and the incidence of myocardial infarction appeared to increase with storage duration. A third of all patients suffered from infections (i.e., surgical site infection or pneumonia), while almost 40% of all patients developed acute respiratory distress syndrome with no particular prevalence towards a particular mean storage duration.

## 4. Discussion

We included patients given massive transfusion, defined by transfusion of 10 or more units of PRBCs within 24 hours. Only about 5% of the estimated annual 50 million trauma cases in the U.S. require this much blood, but they account for 10 to 15% of all transfused blood products [2] and accrue more than 4 billion dollars of health care cost [31]. Blood transfusion is a strong independent predictor of mortality in trauma and nontrauma patients [32], especially in massively transfused patients [33]. Our results are consistent in that mortality was high in our massively transfused patients, many of whom did not even live six hours. Among those who lived and thus qualified for our analysis, a third died during their initial hospitalization.

Two recent meta-analyses evaluated the relationship between PRBC storage duration and long-term mortality, multiple organ failure, in-hospital infections, duration of mechanical ventilation, and respiratory failure requiring ventilator support [34, 35]. The analysis suggested that storage duration did not affect outcome, but the authors noted considerable heterogeneity concerning patient populations, diversity of interventions, and measurement of clinical outcome. In contrast, Wang and colleagues analysed 21 studies including a total of 409,966 patients concluding that older blood is associated with increased mortality [36].

Patients having cardiovascular/CABG surgery are well studied, but both prospective and retrospective data conflict regarding the potential harm of prolonged stored blood products [1, 37–40]. The most recent large prospective trials in cardiac surgery patients [22] and critically ill patients [23] demonstrate no difference in outcomes including the incidence of multiorgan dysfunction syndrome or mortality [22], the incidence of major illnesses, duration of hemodynamic instability, renal or ventilator support, length of stay in the hospital, or transfusion reactions [23]. However, patients in both studies were typically given just one or two units of red cells.

Trauma patients are likely to experience multiorgan failure [24, 41], infection [28], kidney failure [27], pneumonia [42, 43], deep vein thrombosis [41], and death [27, 41, 43, 44]. Risk is presumably largely related to tissue injury, but the need for large amounts of blood products contributes [33].

Massively transfused patients have been underrepresented in previous studies. For example, only 13% of patients in Edgren's database [20] received more than five units of PRBCs, the RECESS trial [22] included only 145 patients who received more than eight units, and only 156 trauma patients in the ABLE trial were given more than four units of blood [23]. Even in the recently published INFORM trial [45] including 30,000 patients randomized to transfusion of short-term or long-term stored PRBCs, the number of trauma patients and massive transfused patients was negligible similar to other studies including even fewer massively transfused patients [24, 28, 42, 43]. Our analysis is the first focusing exclusively on patients who received a minimum of at least ten units of PRBCs within 24 hours, representing transfusion of 8,046 units in just 305 patients. Nonetheless, there was no association between mean storage duration of PRBCs and in-hospital mortality. Nor was there a differential effect amongst trauma and nontrauma patients.

Stored PRBCs undergo progressive structural and conformational changes associated with proposed subsequent worsening of quality, function, and viability of PRBC after transfusion [17, 46, 47]. Assessing effects of blood product age in our patient population receiving at least 10 units of PRBCs is challenging as the majority of patients do not receive exclusively old nor young blood products, but a mixture of both.

**Figure 3:**
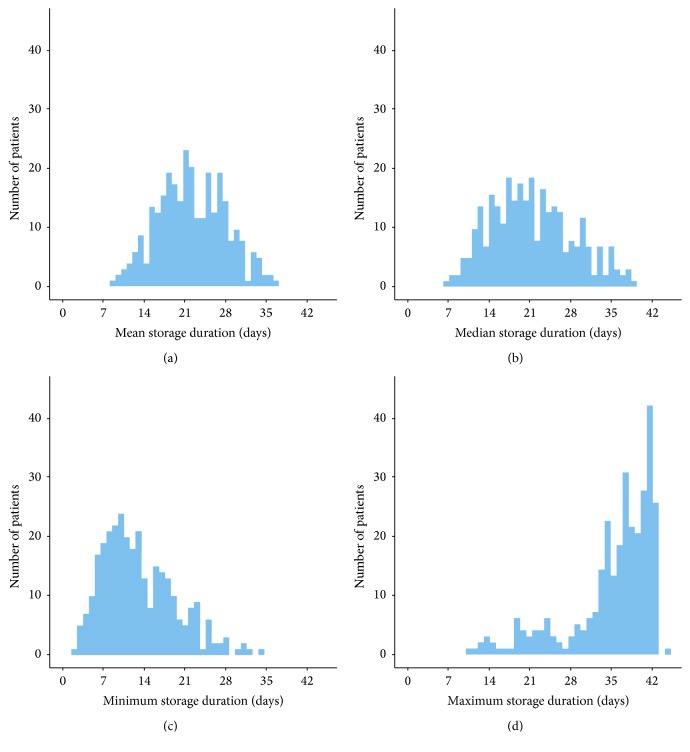
Histograms of the observed values of patient RBC storage duration assembled taking (a) mean, (b) median, (c) minimum, and (d) maximum storage duration as dependent variable. The histogram indicates lack of discrimination between short and prolonged stored blood products.

Unfortunately, no clear cut-off time has been defined after which changes during storage become clinically important with the result that various investigators have used arbitrary storage thresholds (i.e., 14 days [27], 21 days [22], or 28 days [41] of storage time) or based their analyses on the oldest unit [41]. As most blood banks in the U.S. and worldwide follow the inventory principle “first-in-first-out” to avoid outdating of stored blood products [23, 48], massively transfused patients typically receive PRBCs with a considerable range of storage durations because a single patient may use a considerable fraction of the matching units for an entire hospital, even in a major trauma centre like the University of Louisville Hospital. We therefore used mean storage time as a continuous variable, with a consequence that the difference between the first and fourth quartile of PRBC storage duration was only six days. However, histograms displaying the distribution of PRBC storage duration considering median, minimum, and maximum storage duration revealed a similar difference between patients receiving short or prolonged stored products (Figure 3). With the relative broad distribution of storage times of PRBCs/patient, it is not possible to determine whether a specific storage threshold exists (i.e., 35 days [49]) beyond which age will effect outcome in this particular patient population. While this relatively small range limits our ability to assess the specific effects of blood age on mortality, our conclusion that storage duration and mortality are unassociated probably applies broadly.

Mortality within 6 hours after hospital admission increased is doubled in patients who receive 4 or more units of resuscitation fluid [50]. We excluded 36 patients who died within the first 6 hours after starting massive transfusion as most experienced such severe trauma (i.e., penetrating trauma to major vessels and organs with 50% of the patient during initial surgery) that the storage lesion of transfused red cells is unlikely to have caused their demise. In fact, some investigators postulate that some detrimental effects of prolonged red cell storage become evident more than two weeks after transfusion [1]. Our average observation time was 21 days (minimum: 1 day, maximum: 138 days) and therefore remains possible that we missed longer-term transfusion-related outcomes.

As in all observational studies—particularly, those involving emergency surgeries in trauma patients—missing data are a major concern. We reviewed the records of 498 patients who had massive blood transfusions. Despite all efforts, we were unable to retrieve complete data for many patients, a problem that has been noted in previous studies [51]. We therefore only included patients in whom more than 80% of all study-related data were available. A limitation of our analysis is that we cannot determine the effect of missing data in the patients we included, much less the effect of excluding patients who had much missing data.

In summary, our data suggest that the median storage time of PRBCs transfused is not associated with in-hospital mortality amongst patients given at least 10 units of PRBC within 24 hours.

## Figures and Tables

**Table 1:** Summary of baseline patient characteristics by quartiles of patient-specific mean storage duration of transfused red blood cells. Statistics reported as a percentage, mean ± standard deviation, or median [first quartile, third quartile]. Medians and quartiles are reported when the sample skewness coefficient is >0.9 in absolute value.

Factor	Level	First quartile (8.0–17.7 days)	Second quartile (17.7–21.6 days)	Third quartile (21.6–26.6 days)	Fourth quartile (26.6–36.6 days)	Percent missing
		(*N* = 77)	(*N* = 76)	(*N* = 76)	(*N* = 76)	

Year of admission	2006	16	8	5	7	0.7
	2007	13	11	11	5	
	2008	31	26	27	13	
	2009	9	17	23	41	
	2010	16	16	17	16	
	2011	16	22	17	17	

Age		41 ± 18	45 ± 19	48 ± 18	47 ± 18	

Female sex		32	33	28	29	

Body mass index (kg/m^2^)		26 [22,29]	26 [24,32]	26 [23,32]	25 [24,30]	36.7

Patient blood type	A−	8	11	12	7	0.7
	A+	19	30	33	37	
	AB+	0	4	3	7	
	B−	1	3	4	1	
	B+	8	7	8	12	
	O−	13	3	12	4	
	O+	51	43	29	33	

Type of surgery	Trauma	74	73	70	64	0.7
	Cancer	10	7	11	12	
	General	6	9	11	12	
	Vascular	4	9	5	7	
	Gynecological	4	0	4	3	
	Orthopedic	1	1	0	3	

Heart disease		13	20	17	18	

Pulmonary disease		14	11	7	8	

Renal disease		0	5	1	1	

Hypertension		26	39	41	37	

Diabetes mellitus		13	4	16	12	

Carcinoma		10	16	12	16	

Liver disease		9	16	12	13	

Tobacco use		29	29	21	24	

Alcohol use		12	11	11	8	

Illicit drug use		9	7	5	8	

Initial heart rate (beats per minute)		107 ± 35	105 ± 37	102 ± 35	103 ± 32	1.3

Initial systolic blood pressure (mmHg)		108 [82, 122]	100 [84, 123]	101 [88, 123]	111 [87, 130]	0.7

Initial temperature (°C)		36 [36, 37]	36 [35, 37]	36 [36, 37]	36 [35, 37]	14.1

ED crystalloids (L)		2.6 [1.0, 4.0]	2.0 [1.0, 2.6]	1.5 [1.0, 2.3]	2.0 [1.0, 2.5]	32.1

Length of surgery (min)		146 [100, 240]	140 [99, 214]	141 [94, 253]	140 [104, 240]	6.9

Total number of units transfused		23 [17, 38]	23 [16, 35]	22 [17, 31]	18 [15, 25]	

Minimum RBC storage duration		8 ± 3	11 ± 4	14 ± 5	19 ± 7	

Maximum RBC storage duration		33 [20, 37]	37 [32, 40]	37 [34, 41]	39 [36, 41]	

*Initial laboratory values*						
Hemoglobin (g/dL)		10 [8, 12]	11 [8, 12]	11 [9, 12]	10 [9, 12]	4.3
Blood pH		7.2 [7.1, 7.3]	7.2 [7.0, 7.3]	7.2 [7.1, 7.3]	7.2 [7.1, 7.4]	2.3
Platelet count (k/mcL)		190 [120, 227]	180 [94, 256]	187 [111, 248]	184 [100, 264]	2.3
International normalized ratio		1.5 [1.2, 1.9]	1.5 [1.2, 1.9]	1.5 [1.2, 1.8]	1.4 [1.1, 2.0]	4.9
Activated prothrombin time (s)		16 [13, 20]	15 [13, 20]	15 [12, 20]	15 [12, 20]	5.2
Activated partial thromboplastin time (s)		37 [29, 59]	35 [27, 57]	40 [27, 55]	33 [26, 64]	4.9

*Subset of trauma patients*		(*N* = 57)	(*N* = 55)	(*N* = 53)	(*N* = 48)	—

Blunt injury		65	65	64	58	
Penetrating injury		25	25	32	27	
Other type of injury		11	9	4	15	
Injury severity score		30 ± 10	29 ± 14	28 ± 13	27 ± 11	2.8
Glasgow coma score		10 ± 5	9 ± 6	10 ± 5	10 ± 5	

**Table 2:** Summary of baseline patient characteristics by type of surgery. Statistics reported as a percentage, mean ± standard deviation, or median [first quartile, third quartile]. Medians and quartiles are reported when the sample skewness coefficient is >0.9 in absolute value.

Factor	Level	Nontrauma	Trauma	Percent missing
		(*N* = 91)	(*N* = 214)	
Year of admission	2006	2	12	0.7
	2007	10	10	
	2008	29	23	
	2009	27	20	
	2010	16	16	
	2011	15	19	

Age		53 ± 15	42 ± 18	

Female sex		42	26	

Body mass index (kg/m^2^)		25 [23, 31]	27 [24, 30]	36.3

Patient blood type	A−	8	10	0.7
	A+	32	29	
	AB+	4	3	
	B−	1	3	
	B+	7	9	
	O−	12	6	
	O+	36	40	

Heart disease		33	10	

Pulmonary disease		15	7	

Renal disease		5	0	

Hypertension		60	25	

Diabetes mellitus		22	7	

Carcinoma		36	4	

Liver disease		21	9	

Tobacco use		36	21	

Alcohol use		12	9	

Illicit drug use		3	9	

Initial heart rate (beats per minute)		91 [79, 110]	112 [88, 132]	1.3

Initial systolic blood pressure (mmHg)		116 ± 25	98 ± 36	0.7

Initial temperature (°C)		36 [36, 37]	36 [35, 37]	14.1

ED crystalloids (L)		0.6 [0.5, 1.8]	2.0 [1.0, 3.0]	32.1

Length of surgery (min)		214 [120, 400]	130 [90, 206]	6.9

Total number of units transfused		18 [14, 24]	23 [16, 34]	

Minimum PRBC storage duration		15 ± 7	12 ± 6	

Maximum PRBC storage duration		37 [34, 40]	37 [32, 40]	

*Initial laboratory values*				
Hemoglobin (g/dL)		10 [8, 12]	11 [9, 12]	4.3
Blood pH		7.3 [7.2, 7.4]	7.2 [7.1, 7.3]	2.3
Platelet count (k/mcL)		178 [97, 260]	189 [112, 248]	2.3
International normalized ratio		1.3 [1.1, 1.6]	1.5 [1.2, 2.0]	4.9
Activated prothrombin time (s)		14 [12, 17]	16 [13, 20]	5.2
Activated partial thromboplastin time (s)		34 [27, 44]	39 [28, 63]	4.9

**Table 3:** Summary statistics of outcomes by quartiles of patient-specific mean storage duration of transfused red blood cells. Statistics reported as a percentage, mean ± standard deviation, or median [first quartile, third quartile]. Results reflect 305 patients included in the univariable analyses (see Methods). Medians and quartiles are reported when the sample skewness coefficient is >0.9 in absolute value.

Outcome variable	First quartile (8.0–17.7 days)	Second quartile (17.7–21.6 days)	Third quartile (21.6–26.6 days)	Fourth quartile (26.6–36.6 days)	Percent missing
	(*N* = 77)	(*N* = 76)	(*N* = 76)	(*N* = 76)	
*Primary outcome*					
In-hospital mortality	29	43	28	34	
*Secondary outcomes*					
30-day mortality	29	43	25	34	
Duration of hospitalization (d)	21 [7, 34]	15 [4, 30]	20 [8, 32]	14 [4, 26]	
Duration of ICU stay (h)	312 [95, 484]	182 [35, 408]	235 [66, 528]	192 [56, 338]	1
Duration of mechanical ventilation (h)	126 [29, 304]	104 [24, 318]	124 [48, 359]	72 [24, 213]	1.3
Disseminated intravascular coagulopathy	29	14	21	25	
Shock	78	78	82	76	
Sepsis	19	21	20	12	
Wound infection	30	25	29	24	
Arrhythmia	34	38	37	34	
Myocardial infarction	1	4	4	5	
Acute respiratory distress syndrome	39	42	47	38	
Acute kidney injury	14	30	20	17	
Pneumonia	38	33	47	29	
Pulmonary embolism	3	0	8	1	
Transfusion-related acute lung injury	1	1	1	3	

**Table 4:** Independent association between patient-specific mean red blood cell storage duration and secondary outcomes. All estimates are adjusted for age, renal disease, liver disease, initial blood pH, initial antiprothrombin time, and initial activated partial thromboplastin time. Estimates are reported with 95% confidence intervals in parentheses. Analyses reflect the same 259 patients that were included in the multivariable modeling for the primary outcome of in-hospital mortality. The reported measures of association reflect a one-day increase in patient mean red blood cell storage duration.

Outcome variable	Model type	Measure of association	Estimate	*P* value
Duration of hospitalization (d)	Linear regression	Slope (95% CI)	−0.27 (−0.68, 0.13)	0.18
Duration of ICU stay (h)	Linear regression	Slope (95% CI)	−1.79 (−9.82, 6.24)	0.66
Duration of mechanical ventilation (h)	Linear regression	Slope (95% CI)	−0.91 (−7.13, 5.30)	0.77
Disseminated intravascular coagulopathy	Logistic regression	Odds ratio (95% CI)	0.98 (0.93, 1.03)	0.42
Shock	Logistic regression	Odds ratio (95% CI)	1.00 (0.95, 1.05)	0.97
Sepsis	Logistic regression	Odds ratio (95% CI)	0.96 (0.91, 1.02)	0.19
Wound infection	Logistic regression	Odds ratio (95% CI)	0.97 (0.93, 1.02)	0.28
Arrhythmia	Logistic regression	Odds ratio (95% CI)	0.98 (0.93, 1.02)	0.30
Myocardial infarction	Logistic regression	Odds ratio (95% CI)	1.09 (0.96, 1.25)	0.18
Acute respiratory distress syndrome	Logistic regression	Odds ratio (95% CI)	1.00 (0.96, 1.05)	>0.99
Acute kidney injury	Logistic regression	Odds ratio (95% CI)	1.00 (0.94, 1.05)	0.94
Pneumonia	Logistic regression	Odds ratio (95% CI)	0.99 (0.94, 1.03)	0.57
Pulmonary embolism	Logistic regression	Odds ratio (95% CI)	1.10 (0.96, 1.25)	0.15
Transfusion-related acute lung injury	Logistic regression	Odds ratio (95% CI)	1.06 (0.90, 1.26)	0.47
